# Bilirubin as a Therapeutic Molecule: Challenges and Opportunities

**DOI:** 10.3390/antiox10101536

**Published:** 2021-09-28

**Authors:** Christopher A. Adin

**Affiliations:** Department of Small Animal Clinical Sciences, University of Florida, Gainesville, FL 32610, USA; adinc@ufl.edu

**Keywords:** bilirubin, antioxidant, ischemia-reperfusion, Gilbert’s Syndrome, nanoparticle, synthetic

## Abstract

There is strong evidence that serum free bilirubin concentration has significant effects on morbidity and mortality in the most significant health conditions of our times, including cardiovascular disease, diabetes, and obesity/metabolic syndrome. Supplementation of bilirubin in animal and experimental models has reproduced these protective effects, but several factors have slowed the application bilirubin as a therapeutic agent in human patients. Bilirubin is poorly soluble in water, and is a complex molecule that is difficult to synthesize. Current sources of this molecule are animal-derived, creating concerns regarding the risk of virus or prion transmission. However, recent developments in nanoparticle drug delivery, biosynthetic strategies, and drug synthesis have opened new avenues for applying bilirubin as a pharmaceutical agent. This article reviews the chemistry and physiology of bilirubin, potential clinical applications and summarizes current strategies for safe and efficient drug delivery.

## 1. Introduction

For centuries, bilirubin has been known for its contribution to kernicterus, a condition that involves a toxic accumulation of the yellow pigment in the central nervous system of newborn infants [1]. Laboratory studies in the 1950s and 1960s characterized the chemistry of bilirubin in biological fluids, while scientists scrambled to find ways to eliminate this harmful waste product from ailing patients using phototherapy [2,3,4]. It was not until the 1980s that researchers discovered that the story was not so simple: bilirubin was toxic at high doses, but within the normal physiologic range this molecule serves as the most powerful antioxidant in the serum [5]. In fact, an enzyme preserved throughout all eukaryotic organisms converts the water-soluble, non-toxic biliverdin molecule into the insoluble and potentially toxic bilirubin molecule, suggesting that there is a purpose for bilirubin that is essential to most life forms [6,7]. Over the past 50 years, evidence has confirmed that bilirubin plays a key role in cardiovascular health, [8,9,10,11] stroke, [12] diabetes, [13] and metabolic syndrome, [14,15] making the manipulation of serum bilirubin levels a potential therapeutic target for a wide variety of medical conditions. In this article, we will review the evidence for the therapeutic effects of bilirubin in health sciences and we will tackle the difficult question of how to administer this potential therapy to patients in the modern hospital setting.

## 2. Evidence for the Protective Effects of Bilirubin

Drug development involves a high failure rate as candidate small molecule drugs and biologics must be tested through an ascending pathway that begins with in vitro experiments, then pass through rodents and large animal models before entering human clinical trials. Since bilirubin is a natural molecule that exists in all eukaryotes, any positive or negative effects in human and animal models can be investigated through epidemiologic studies that relate serum bilirubin concentrations to health outcomes. In fact, a mutation in the gene for uridine diphosphate glucuronosyltranferase (UGT-1A), the enzyme responsible for conjugation of bilirubin, is prevalent in the human population and approximately 5–10% of the population have mildly elevated serum total bilirubin concentrations of 1–6 mg/dL (17–100 µM) due to a condition termed Gilbert’s Syndrome [16]. Patients with Gilbert’s Syndrome have an insertion in the promoter region of the gene, leaving the gene functional, but causing reduction of gene transcription to ~20% of normal [17]. Many patients with Gilbert’s Syndrome can have concurrent genetic abnormalities affecting bilirubin metabolism at multiple levels, including the genes for heme oxygenase-1, and biliverdin reductase [18]. An animal model for Gilbert’s Syndrome has been identified in Gunn rats, providing a correlate for experimental studies of hyperbilirubinemia [19]. Decades of epidemiologic studies in people with Gilbert’s Syndrome have shown that mild elevations in serum bilirubin concentrations cause a decreased risk for coronary artery disease, stroke, myocardial infarction and improve outcomes in organ transplantation, with virtually no pathologic effects noted in affected individuals [10,12,15,16,17,18,20,21,22,23,24,25,26,27,28]. Patients with the more rare Criggler-Najar Syndrome have a mutation in the coding section of the UDPGT1A1 gene that causes complete inactivity of the UGT-1A enzyme, resulting in serum bilirubin concentrations in the 20–45 mg/dL 425–765 µM) range [17]. At this level, toxic effects of bilirubin are observed, including potentially fatal neurotoxicity prior to adulthood unless liver transplantation is performed [17]. These observational studies have been extremely useful in providing much of the needed safety, dose response and efficacy studies in human beings that would be gleaned from clinical trials.

## 3. Bilirubin Chemistry

**Figure 1:**
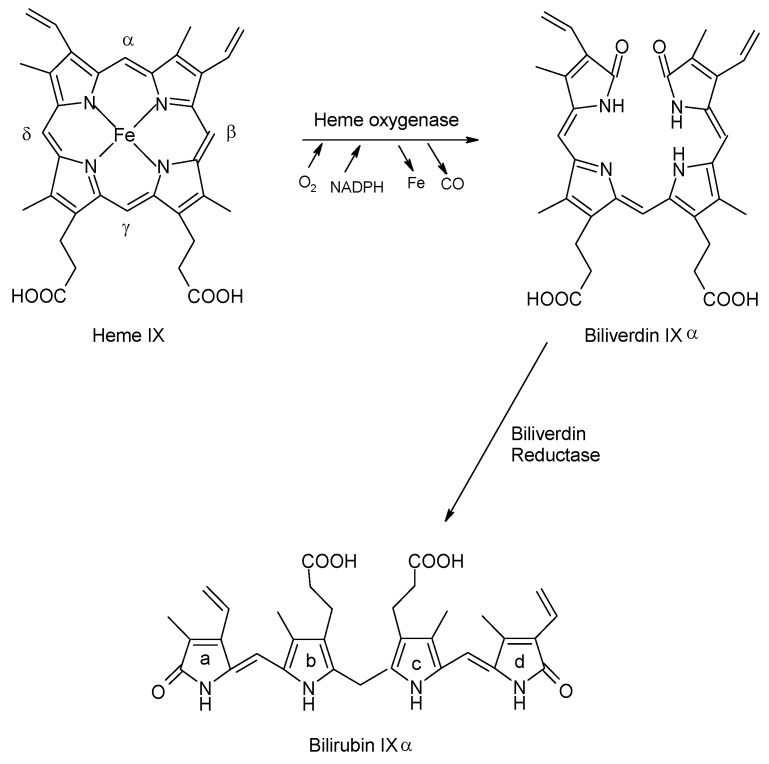
Degradation of Heme and Production of Bilirubin: Heme is cleaved by the enzyme heme-oxygenase at the α-meso carbon bridge, producing equimolar amounts of biliverdin, carbon monoxide and iron. Biliverdin, a water soluble and relatively non-toxic molecule, is then converted into bilirubin through the actions of biliverdin reductase. (From Kirkby K.A. and Adin C.A., Products of heme oxygenase and their potential therapeutic applications, *Am. J. Physiol. Renal Physiol.* **2006**, *290*, F563–F571, doi:10.1152/ajprenal.00220.2005, [6]).

**Figure 2:**
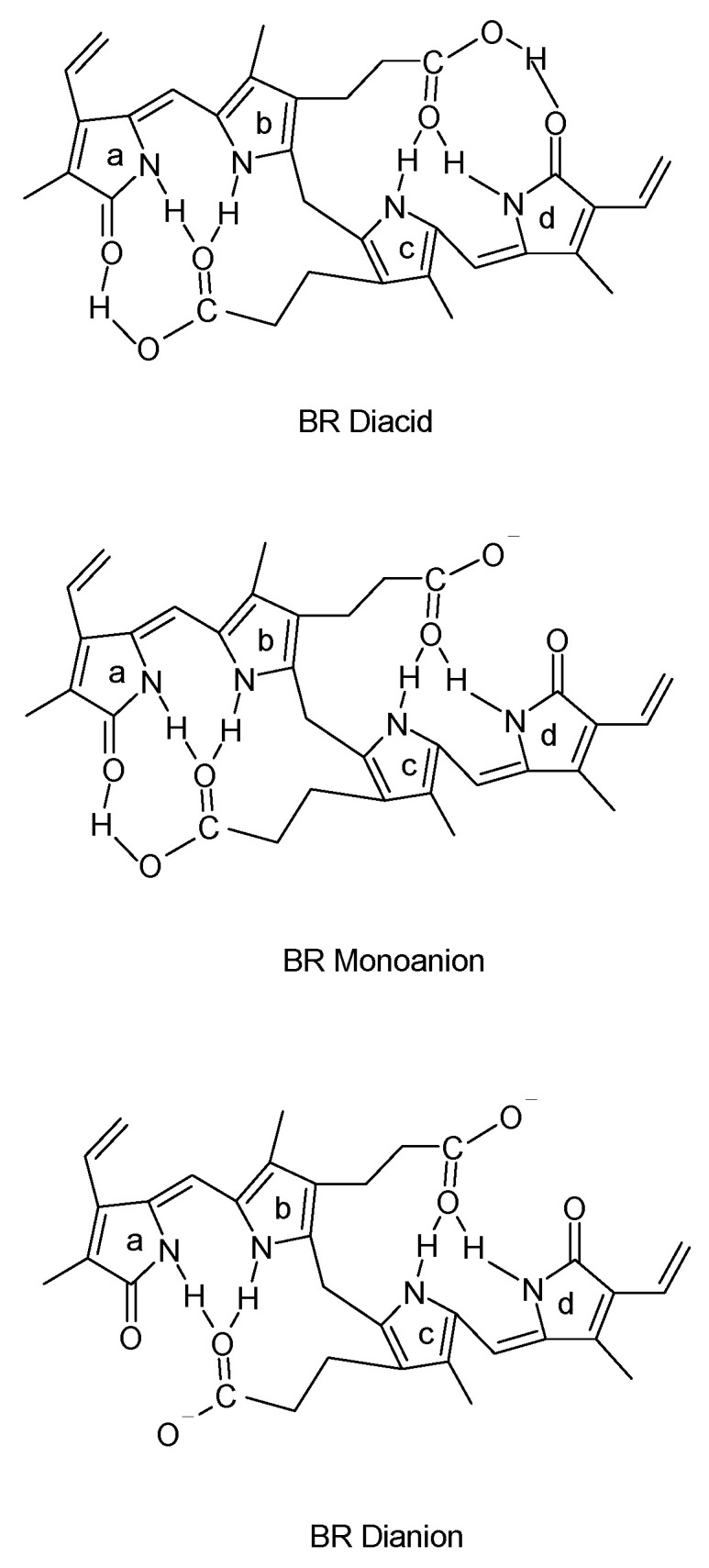
Molecular Forms of Bilirubin at Various pH Levels: Bilirubin is present as a diacid, also known as Bilirubin IXα, with small amounts of the monoanion and dianion also present at physiologic pH (7.4). Elevating the pH by adding sodium hydroxide will encourage the formation of the diacid, increasing the solubility of bilirubin. In the serum, solubility at neutral pH is improved by protein binding to albumin (from Kirkby and Adin, Products of heme oxygenase and their potential therapeutic applications, *Am. J. Physiol. Renal Physiol.* **2006**, *290*, F563–F571, doi:10.1152/ajprenal.00220.2005, [6]).

Bilirubin is produced as a result of the catabolism of the large heme (Fe-protoporphyrin IX) molecule by the enzyme heme oxygenase (HO). While heme is a component of many intracellular enzymes, the majority is derived from the catabolism of hemoglobin that is released daily in the spleen and liver during the scavenging of senescent erythrocytes. Heme oxygenase cleaves the heme molecule at the α-meso carbon bridge, producing equimolar amounts of biliverdin, carbon monoxide and iron (Figure 1). The relatively non-toxic, water-soluble biliverdin molecule is then reduced by the enzyme biliverdin reductase to form the less soluble and more toxic bilirubin IXα, a likely evolutionary adaptation to produce this important cytoprotectant [29]. Bilirubin is a tetrapyrrole that is composed of two rigid dipyrroles joined by a methylene bridge at carbon 10. Of the three isomers that exist (IIIα, IXα, and XIIIα), IXα is the natural form [6,30]. The pK_a_ of the -COOH groups on the carboxymethyl sidechains of the bilirubin molecule are 8.1 and 8.4, causing it to exist primarily as a protonated diacid at neutral pH (Figure 2). Internal bonding of the polar groups at neutral pH results in the relatively low water solubility of bilirubin (~70 nM). In vitro, raising the pH above 9.5 by the addition of NaOH will markedly increase the solubility of bilirubin to >60 mM [30]. In the plasma, the solubility of bilirubin at physiologic pH (7.4) is increased through a different mechanism, with >99% of bilirubin being bound to multiple binding sites on the albumin molecule [30]. The small amount of unconjugated and unbound bilirubin in the plasma is taken up by hepatocytes by passive diffusion through the cell membrane, binds to intracytosolic proteins and is conjugated with glucuronic acid by the enzyme UDPGT [31]. This water-soluble form of bilirubin is excreted into the intestine with bile and is partially eliminated in the feces.

## 4. Physiology

Normally, bilirubin levels are tightly regulated in healthy adults, with concentrations ranging from 5 to 15 µM in the serum. Pathologic alterations in serum bilirubin levels during disease states can be classified as pre-hepatic (hemolysis), hepatic (liver failure) or post hepatic (biliary obstruction). Prior to the recognition of Rh incompatibilities, neonatal hyperbilirubinemia, or jaundice, was a common condition that resulted from massive hemolysis. Even now, one in ten children is affected by neonatal jaundice with serum bilirubin concentrations in excess of 290 µM [32]. Much of the understanding of the toxic effects of bilirubin was obtained through observations of babies that suffered from kernicterus, a central nervous system disorder that results from the neurotoxic effects of bilirubin on the brain. Due to its lipophilic structure, free bilirubin can pass through the blood–brain barrier. While this process is slow in adults, the blood–brain barrier of neonates is particularly permeable to bilirubin in the first days of life, and hyperbilirubinemia contributes to vascular dysfunction that causes additional susceptibility to bilirubin-induced neurotoxicity [32]. Once bilirubin enters the neuron, cell death can be induced by both necrosis and apoptosis. Oddly, while bilirubin is an antioxidant at low doses, high levels of unconjugated bilirubin cause oxidative stress in neurons through inhibition of cytochrome-c oxidase activity and increased production of the superoxide anion [33]. Apoptosis is also induced through disruption of the mitochondrial membrane, release of cytochrome-c and subsequent activation of caspase-3 [34].

Smaller, non-pathologic elevations in bilirubin concentrations occur in healthy individuals due to upregulation of the HO system in response to stress or extreme exercise. Heme oxygenase consists of two isoforms, the inducible HO-1, and the constitutively expressed HO-2 [6]. HO-2, which is in the highest concentrations in the brain, liver, and testis, is responsible for heme catabolism under normal conditions [35]. The inducible form of the enzyme, HO-1, is a heat-shock protein that is triggered by tissue injury and oxidant stress associated with ischemia/reperfusion, hypoxia, and inflammatory conditions. Upregulation of HO-1 has been shown to produce increased local tissue concentrations of bilirubin, but levels remain well within the physiologic range (<15 µM) and do not contribute to bilirubin toxicity [36].

## 5. Mechanisms of Action

### 5.1. Antioxidant Activity

Bilirubin has powerful antioxidant activity and has been shown to be capable of protecting from 10,000 fold molar excess of hydrogen peroxide [37,38]. Reduction of oxidant-related stress is the key mechanism behind the protective effects of bilirubin in the kidney, heart and brain, [39,40,41,42,43] the three organs that are most commonly affected by ischemia/reperfusion during cardiac bypass, myocardial infarction, stroke and severe hypotension. Bilirubin has similar effects in preventing nitrosative injury that is induced by peroxynitrite, a potent oxidant that is formed by the combination of nitric oxide (NO) and the superoxide anion, as occurs during renal ischemia reperfusion injury [44,45,46]. Elevated bilirubin levels in humans with Gilbert’s Syndrome were shown to inhibit protein and lipid peroxidation when challenged with hypochlorous acid, suggesting a potential mechanism for the prevention of atherosclerosis in these individuals [47].

### 5.2. Anti-Inflammatory and Immunomodulatory

**Figure 3:**
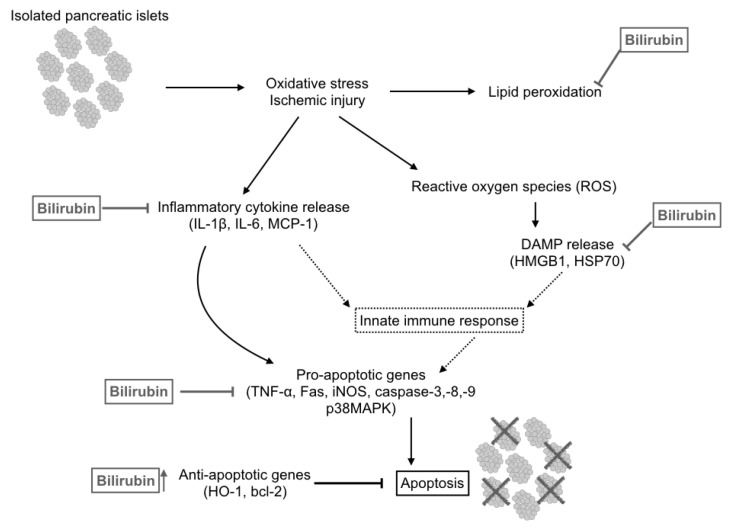
Cytoprotective Effects of Bilirubin: Protective effects of bilirubin occur at multiple levels on the pathway to apoptosis. This schematic depicts some of the demonstrated effects of bilirubin on transplanted pancreatic islet cells. (From Fullagar, B.; Rao, W.; Gilor, C.; Xu, F.; He, X.; Adin, C.A. Nano-Encapsulation of Bilirubin in Pluronic F127–Chitosan Improves Uptake in β Cells and Increases Islet Viability and Function after Hypoxic Stress. *Cell Transplant.* **2017**, *26*, 1703–1715, doi:10.1177/0963689717735112. [53]).

After these early studies, investigators began to discover that bilirubin has immunomodulatory effects, with particular focus on decreasing the release of damage associated molecular patterns (DAMPS) and IL-1 β by injured pancreatic islet cells, which are key initiators of the innate immune response after cell or organ transplantation [48]. Through suppression of these early signals, bilirubin supplementation has even enabled the generation of tolerance to organ transplants, reducing apoptosis, and release of inflammatory mediators (IL-1 β, soluble intercellular adhesion molecule 1 [ICAM1], and monocyte chemoattractant protein 1 [MCP-1], eliminating the early signaling that leads to allo-recognition and acute or chronic graft rejection [49]. Cytoprotective effects of bilirubin are enacted in many ways beyond its direct anti-oxidant activity, including the inhibition of pro-apoptotic genes (TNF-α, Fas, iNOS, Caspase-3,-8,-9, and p38MAPK) and upregulation of antiapoptotic genes (HO-1 and bcl-2) (Figure 3) [49,50,51,52,53]. Bilirubin and biliverdin supplementation have also been shown to prevent pro-inflammatory signaling through inhibition of NF-κB regulated pathways in models of pancreatic, bowel, and cardiac injury [54,55,56]. In vitro experiments with neutrophils from patients with neonatal hyperbilirubinemia have demonstrated that bilirubin has a direct anti-inflammatory effect on LPS stimulated neutrophils, reducing IL-8 and macrophage inhibitory protein-1β while increasing antioxidant enzymes such as superoxide dismutase and HO-1 [57]. The anti-inflammatory and immunomodulatory effects of bilirubin are also integral to the anti-atherosclerotic effects seen in patients with Gilbert’s Syndrome [20]. Bilirubin has been shown to prevent endothelial cell injury at multiple levels by both inhibiting the release of superoxide from neutrophils, and by directly scavenging superoxide [58,59]. Similarly, bilirubin reduces P-selectin expression, thereby reducing leukocyte adherence and migration that contributes to atherosclerosis [60].

### 5.3. Hormone-Like Effects and Signaling

While the beneficial cardiovascular effects of mildly elevated serum bilirubin concentrations have been known for several decades, recent observations have shown that people with low bilirubin levels are more likely to suffer from metabolic syndrome and diabetes [13,15,21]. Supplementation of bilirubin can ameliorate hepatic steatosis, reduce ketosis, and improve fat utilization in a diet-induced rodent model of non-alcoholic fatty liver disease and obesity [61]. Knockout of biliverdin reductase in adipocytes leads to oxidative stress, lipid accumulation, hypertrophy and reduced mitochondria in those cells [62,63]. Through a series of elegant studies, bilirubin has been found to be an important signaling molecule that is a direct ligand for peroxisome proliferator activated receptor alpha (PPARα) and that 95% of bilirubin-controlled gene activity in HepG2 hepatocytes is PPARα dependent [64,65,66]. Bilirubin exerts its PPARα-mediated metabolic effects in both hepatocytes and in adipocytes and has been shown to have no interaction with PPARγ or δ [67]. End effects of PPARα stimulation by bilirubin include increased expression of Cpt-1, UCP-1, and Adrb3, leading to fat-burning, weight-loss, and improved glucose homeostasis [15].

## 6. Potential Therapeutic Applications

### 6.1. Cardiovascular Disease

**Figure 4:**
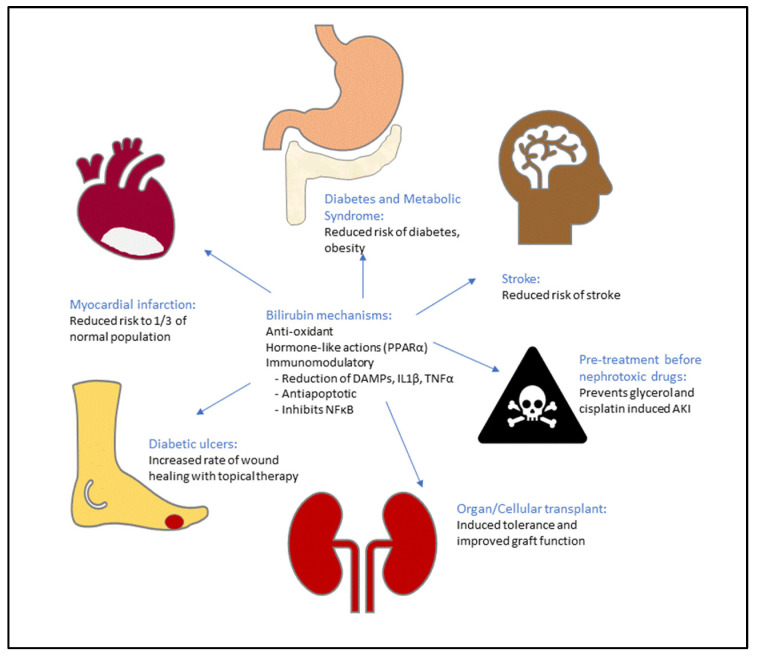
Potential Clinical Applications of Bilirubin: mild elevations of bilirubin concentration in the 17–100 µM protect individuals with congen-ital hyperbilirubinemia against many of the top causes of mortality, while supplementation of bilirubin to similar levels has recapitulated these results in animal models.

Observational studies have provided incontrovertible evidence that human populations with Gilbert’s Syndrome are protected against myocardial infarction, atherosclerosis, and stroke [12,16,20,47,68,69,70]. Given that cardiovascular disease is the leading cause of mortality globally and that Gilbert’s Syndrome decreases the risk of cardiovascular mortality to 1/3 that of the general population [71], leveraging the effects of mild hyperbilirubinemia has enormous potential to impact human health. Mechanisms for this protection are likely to occur at multiple levels, including anti-atherosclerotic effects through inhibition of lipid oxidation, improved tolerance of cardiac ischemia-reperfusion injury, reduced platelet adhesion, and lowering lipid levels [20,47,72,73,74,75]. Experimental models of cardiac ischemia-reperfusion injury have confirmed these findings in rats with either congenital hyperbilirubinemia due to UGT deficiency, or in those that received supplemental bilirubin prior to the insult. Bilirubin (10 mg/kg) administered intraperitoneally prior to occlusion of the left anterior descending coronary artery led to a decrease in the infarct area and improved function during the period of ischemia [76]. In a similar experiment using the Gunn rat, chronically elevated bilirubin levels improved stress resistance, coronary flow and reduced infarct area after experimentally induced ischemia-reperfusion injury [19]. While there is little debate about the cardiovascular benefits of bilirubin, the challenge in applying this molecule as a therapeutic agent is the need to maintain mild elevations in serum bilirubin concentrations over the lifetime of at risk human patients, essentially creating iatrogenic Gilbert’s Syndrome in a large portion of the population (Figure 4).

### 6.2. Acute Kidney Injury

Aside from the protective effects of bilirubin in renal ischemia-reperfusion injury, this molecule appears to provide a separate and important protective effect against toxin-induced acute kidney injury (AKI), which is a significant cause of morbidity and mortality in human patients. Studies in rodent models have shown that iatrogenically induced cholestasis protected against glycerol induced renal injury in rats [77]. In a study that had direct implications on human health, Gunn rats with mild elevations in serum bilirubin concentration were shown to have complete protection from cisplatin-induced AKI, without affecting the anti-neoplastic effects of this key chemotherapy agent [78]. Based on these rodent studies, there is excellent potential to use bilirubin or related molecules as a “pre-treatment” before administration of nephrotoxic drugs, including chemotherapy agents, aminoglycoside antibiotics, and others, opening up the more widespread use of these highly effective agents that currently have a narrow therapeutic range.

### 6.3. Transplantation

A significant body of work has been documented that bilirubin augmentation can improve outcomes in nearly every form of organ and tissue transplantation in rodents, including small bowel [55], heart [79], pancreatic islet [48], and liver transplants [80]. Through attenuating tissue injury during ischemia and reperfusion injury that is associated with the process of organ or cellular transplantation, bilirubin or biliverdin infusion prior to implantation has been shown to induce immune tolerance in both islet and cardiac allografts by suppression of the innate immune response [48,56,81]. In lieu of systemic administration, a simple bilirubin rinse of cardiac allografts prior to reperfusion in a rat model ameliorated ischemia reperfusion injury, improving function and decreasing apoptosis [79]. While no studies have involved the administration of exogenous bilirubin in human transplant recipients, naturally occurring increases in serum bilirubin concentrations are associated with improved outcomes in kidney and liver transplants, while lower levels were associated with graft loss and all-cause mortality in human kidney transplant recipients. [24,25,28] Due to the planned nature of transplant procedures and the ability to administer bilirubin to the recipient immediately prior to surgery or to the transplant tissue as an additive to preservation solutions, organ transplantation is one of the most practical areas for the application of bilirubin as a therapeutic molecule.

### 6.4. Diabetes and Metabolic Syndrome

Serum bilirubin concentration has been inversely associated with risk for diabetes [13,15,22] and metabolic syndrome [21]. Serum bilirubin has also been identified as a predictor of new onset type-2 diabetes mellitus in at-risk patient populations [26]. It was only recently that these effects were explained, as bilirubin was discovered to have direct hormonal activity by serving as a ligand for the peroxisome proliferator-activated receptor alpha (PPAR-α), one of the key regulators of metabolism [65,66,67]. Patients with higher serum bilirubin concentrations tend to have leaner body mass, with a higher proportion of “brown fat”, which is more metabolically active, while patients with low serum bilirubin have a higher “white fat” content, which predisposes to obesity and metabolic syndrome [15]. In subjects with existing diabetes, serum bilirubin concentration also impacts the likelihood of experiencing diabetes related medical problems, such as diabetic retinopathy and vascular disease [23,82,83]. With the widespread increase in the incidence of obesity, T2DM and metabolic syndrome in the United States, this area presents similar challenges and opportunities as described for cardiovascular diseases: strategies to achieve mild increases in serum bilirubin across large portions of the population could have a marked impact on human health.

### 6.5. Non-Healing Wounds

Due to the cytoprotective and immunomodulatory effects of bilirubin, the potential for its use as a topical agent for wound healing has been investigated. In particular, chronic, non-healing wounds have been found to show evidence of increased oxidative stress which, in combination with increased activity of inducible Nitric Oxide Synthetase, produces conditions that increase the formation of peroxynitrite and other reactive nitrogen species [84]. In rat models that involved wounds created both healthy [85] and diabetic rats [86,87], application of topical bilirubin caused a significant decrease in wound size compared to controls. Positive effects of topical bilirubin on wound healing were correlated with reduced oxidative activity and with modulation of the inflammatory wound environment by down-regulation of Interleukin-1β, TNF-α and intercellular adhesion molecule-1, and upregulation of interleukin-10. [85,86,88] Vascular endothelial growth factor, collagen deposition and overall wound healing rate were also increased in wounds treated with a topical bioadhesive hydrogel containing bilirubin/betacyclodextrin combination when compared to bilirubin alone [87]. Given the positive effects in animal models and the relatively low risk of systemic toxicity with topical administration, chronic wounds may present an opportunity for early application of bilirubin in clinical patients.

## 7. Therapeutic Dose Range

Studies in animal models have shown that augmentation of bilirubin levels to achieve concentrations between 0.5 and 200 µM can be effective in a wide variety of tissues and injury types. Evidence would suggest that therapeutic concentrations of bilirubin appear to be related to experimental design. Studies using cell culture models and perfused organs show a narrow therapeutic range, with micromolar doses in the 10–20 µM range being most effective, but concentrations above 25 µM induced apoptosis [89,90]. For example, studies in the isolated-perfused kidney showed that protection of renal function and histology was best achieved at a concentration of 10 µM in the perfusate [91]. Interestingly, studies of HO induction in the kidney parallel these findings and suggest that 10 µM is the tissue concentration of bilirubin that is achieved at 3 h after HO induction, at a time when maximal protection is demonstrated [36]. In a rat liver transplantation model, flushing of the graft with 5 µM bilirubin was maximally protective on liver function post-transplant, with doses above 5 µM resulting in a decrease in function. Pancreatic islet transplant studies involving supplementation of cell culture media with exogenous bilirubin have demonstrated that maximal protective effects from hypoxia-induced oxidative injury are seen at 10–20 µM [48].

In contrast, models involving systemic administration of bilirubin to live animals have used higher doses to show beneficial effects with less evidence of toxicity. In a rat cholestasis model, ligation of the common bile duct provided marked protection from glycerol induced kidney injury, with bilirubin concentrations averaging 148 µM at the time of injury [77]. Another model used wild type (Wistar), heterozygous Gunn and homozygous Gunn rats with heritable hyperbilirubinemia in a cisplatin-induced nephrotoxicity model [78]. In this study, mild bilirubin elevations in the heterozygous Gunn rat (6.8 µM) were partially protective, but moderate hyperbilirubinemia (71 µM) seen in homozygous Gunn rats provided complete protection. Observations in human subjects with Gilbert’s Syndrome align with these experimental observations in Gunn rats, with serum bilirubin concentrations of >17 µM conveying profound protective effects against cardiovascular disease in homozygotes [71]. The wider therapeutic range and lack of apparent toxicities seen in “whole animal” studies may be related to the physiology of bilirubin at the organism level, where the blood–brain barrier, protein binding, glucuronidation and excretion in the feces all contribute to regulation of bilirubin levels in susceptible tissues.

A small number of studies have attempted to use parenteral doses of exogenous bilirubin ranging from 3 to 20 mg/kg to induce hyperbilirubinemia in animal models. Intravenous administration of 15 mg/kg of exogenous bilirubin followed by infusion of 30 mg/kg over 75 min achieved serum levels of 13 mg/dL (220 µM) and ameliorated intestinal reperfusion injury through antioxidant effects, with no apparent harm in controls receiving bilirubin alone [92]. A similar dosing protocol minimized histologic injury, but failed to preserve renal function in rats undergoing renal ischemia-reperfusion injury [93]. Lastly, cardiac ischemia/reperfusion injury was partially ameliorated in Wistar rats that were administered 10 mg/kg bilirubin intraperitoneally before ligation of the left anterior descending coronary artery [76].

## 8. Delivery Methods

### 8.1. HO-1 Induction

Recognizing that the HO-1 enzyme system is a natural protective mechanism, upregulation of HO-1 expression has been proposed as a method to achieve cytoprotection [35]. HO-1 induced heme catabolism leads to the release of iron, carbon monoxide, and biliverdin, which is then converted into bilirubin through the actions of biliverdin reductase. HO-1 expression can be upregulated through pharmacologic means by administering the substrates for the reaction (e.g., hemoglobin or myoglobin), or by stimulating with a planned injury in a process called preconditioning. Clearly, this approach is only useful in instances when organ injury can be anticipated and pre-treatment is possible, as in planned cardiac ischemia for bypass procedures or in organ transplantation. Furthermore, while effective methods for preconditioning have been identified, each has the potential for causing patient harm (e.g., periodic organ ischemia prior to placing clamps for cardiac bypass) and physicians are wary to use them. Several pharmacologic and neutraceutical strategies for HO-1 induction are being pursued [94], but until a non-harmful chemical inducer HO-1 expression is discovered, this method is unlikely to be widely applied in human patients.

### 8.2. Biliverdin Administration

Administration of biliverdin as a water-soluble and less toxic precursor to bilirubin has been considered as a method to provide cytoprotection as biliverdin is rapidly converted into bilirubin by the enzyme biliverdin reductase [37,52,55,56,95]. Baranano proposed that bilirubin is reduced to biliverdin after exposure to free radicals, and that recycling of bilirubin can then be achieved through the actions of biliverdin reductase, creating a powerful redox cycle that exponentially increases the antioxidant activity of the system [37]. This mechanistic explanation was later questioned when analysis failed to show that biliverdin was produced after exposure of bilirubin to hydrogen peroxide radicals [38]. Until recently, commercially available biliverdin was exclusively animal-derived, providing little advantage over the use of bilirubin. However, one team has shown that scalable production of biliverdin can be achieved using genetically modified *E. coli*, creating a potential method to achieve a safe and non-toxic pharmaceutical form that could be administered as a “pro-drug” to increase bilirubin concentrations in tissues, organs or patients that are about to undergo planned hypoxia or ischemia reperfusion injury [96].

In a related approach, another line of investigation has explored the manipulation of biliverdin reductase activity as a therapeutic target for increasing serum bilirubin levels [97]. These investigators theorize that the antioxidant activity of bilirubin is exponentially increased through a proposed redox cycle involving the reversible conversion of biliverdin into bilirubin [37]. This assertion is supported by the improved antioxidant activity and resistance to metabolic syndrome and diabetes in both rodents and humans with increased biliverdin reductase activity and suggests a potential target for pharmaceutical action or genetic manipulation [62,98]. To date, there are no practical methods to achieve this goal in patients that do not have naturally increased activity of biliverdin reductase.

### 8.3. Inhibition of Bilirubin Conjugation by UGT

Using their understanding of bilirubin physiology, several researchers have proposed methods that could increase local or systemic bilirubin levels by manipulating the pathways for bilirubin conjugation, as occurs naturally in patients with Gilberts syndrome. Early methods to achieve UGT inhibition included the use of existing pharmaceutical agents that had been noted to inhibit hepatic glucuronidation, such as probenecid or rifampin [99]. Unfortunately, each of these drugs comes with the potential for harm. One group has performed a clinical trial using the drug atazanavir in 16 human patients with type-2 diabetes mellitus [83]. Atazanavir is an antiviral agent that is used in the treatment of human immunodeficiency virus, but has a side effect of causing mild elevations in serum bilirubin (64 µM) by inhibiting UGTA1A activity [100]. After only 3 days of treatment, atazanavir induced increases in unconjugated bilirubin levels that improved plasma antioxidant capacity, and improved endothelium dependent vasodilation in diabetics [83]. The recent discovery that UGT activity was controlled by phosphorylation has led another group to describe a rapid, reversible method to down-regulate UGT activity using either curcumin or calphostin-C, a protein-kinase C inhibitor [101]. While this method was only described in cell culture, there is the potential for future application in human patients.

### 8.4. Natural Bilirubin Administration

Given the aforementioned challenges associated with HO-1 induction, direct supplementation of exogenous bilirubin has been one of the primary approaches used in animal and cell culture models. While exogenous bilirubin has been shown to be effective in the majority of these studies, there are a number of challenges to be overcome before this therapy can be translated into medical practice. To date, the products used in research studies are chemical grade and have been universally animal-derived, carrying the potential for transmitting prions or viral infectious agents. Commercially available bilirubin is porcine in origin and is composed of mixed isomers, and in its current form, it is unsuitable for use in human subjects [102]. Aside from this first and most significant hurdle, there are a number of practical challenges to the use of bilirubin as a therapeutic agent related to the chemistry of this molecule, which makes it insoluble in aqueous solutions at neutral pH. Dissolution of bilirubin powder requires the addition of sodium hydroxide to achieve a pH >9.5, or the use of an organic solvent such as dimethyl sulfoxide (DMSO), leading to problems identifying a vehicle that is appropriate for intravenous administration to patients. Once dissolved, bilirubin is highly protein-bound in plasma, and has poor bioavailability, necessitating repeated intravenous or intraperitoneal administration to maintain therapeutic levels [93].

### 8.5. Nanoparticle Encapsulation

Given the struggles with administering exogenous bilirubin by intravenous administration, nanoparticle (NP) encapsulation has been explored as a method for drug delivery [53,54,61,103]. Encapsulating drugs in polymeric NP can allow targeting of certain cell types based on rates of endocytosis and are particularly applicable in improving the loading capacity for water-insoluble drugs. The enhanced permeability and retention (EPR) effect describes the accumulation of nanoparticles in areas of inflammation or cancer and is based on particle size and allows targeted local drug delivery. Furthermore, manipulations of the composition of the NP can tune the rate of drug delivery. Chitosan is a natural cationic polysaccharide that forms spontaneous micelles with a hydrophobic core, allowing encapsulation of a hydrophobic drug inside the hydrophobic core, while the exterior surface is hydrophilic and compatible with aqueous solutions. Coating chitosan nanoparticles with Pluronic F127, another amphiphilic molecule approved by the Food and Drug Administration for use in pharmaceuticals, allowed improved uptake of bilirubin into pancreatic islet cells and conferred protection against hypoxia-induced injury [53]. Another group has used bilirubin encapsulated silk fibroin nanoparticles for targeted delivery of bilirubin to the inflamed pancreas, where bilirubin was released by trypsin mediated cleavage of the silk fibroin and protected against acute pancreatitis in a rat model [103]. Similarly, nanoparticles assembled from polyethylene glycol (PEG) conjugated bilirubin accumulated in pancreatic islet grafts by the EPR effect, significantly extending the survival of islet allografts through anti-inflammatory and ant-oxidant effects [61].

### 8.6. Synthetic Analogues

While nanoparticle encapsulation technology can aid in the delivery of the hydrophobic bilirubin molecules to certain cell types, the inherent safety problems associated with the use of this animal derived compound are not addressed with this approach. The use of a synthetic form of bilirubin would be ideal, although synthesis of this complex tetrapyrrole molecule would be extremely challenging. Instead, researchers have described to synthesis of bis-pyrroles, molecules which contain two purine rings, while preserving the functional components that lead to anti-oxidant activity. Bilirubin scavenges free radicals through the donation of a hydrogen atom attached to the C-10 bridge, forming a carbon-centered radial [5]. The creation of a bis-pyrrole bilirubin analogue yielded similar anti-oxidant characteristics to naturally derived bilirubin and provided superior protection of pancreatic islet cells subjected to hypoxia induced injury [104]. A related approach is the use of biomimetics, such as cyanobacterial phycobilins, as bilirubin analogues that could convey similar properties [105]. Since the synthetic analogues of bilirubin have similar chemical structures they are also likely to be poorly insoluble in water, and can convey cytotoxic properties at higher concentrations [104].

## 9. Conclusions

Elevation of free bilirubin levels has been shown to convey significant beneficial effects against several of the top causes of global mortality, including cardiovascular disease, diabetes and metabolic syndrome. Acute pre-treatment with bilirubin has also shown success preventing organ and cellular injury during cardiac bypass, organ transplantation or after administration of potentially toxic chemotherapy or antimicrobial drugs. However, the chemical properties of bilirubin and the difficulty of synthesizing this complicated molecule have previously limited its application as a pharmaceutical agent. Recently developed techniques including polymeric nanoparticle drug delivery, synthesis of bilirubin analogues, or the application of genetically engineered bacteria as bioreactors for the generation of porphyrins have overcome many of the barriers to treatment. With these methods in hand, this natural antioxidant and signaling molecule may now be applied to a wide variety of acute and chronic health conditions.
